# Apocynum Tablet Protects against Cardiac Hypertrophy via Inhibiting AKT and ERK1/2 Phosphorylation after Pressure Overload

**DOI:** 10.1155/2014/769515

**Published:** 2014-06-29

**Authors:** Jianyong Qi, Qin Liu, Kaizheng Gong, Juan Yu, Lei Wang, Liheng Guo, Miao Zhou, Jiashin Wu, Minzhou Zhang

**Affiliations:** ^1^Intensive Care Laboratory, Guangdong Province Hospital of Chinese Medicine, 2nd Affiliated Hospital of Guangzhou University of Chinese Medicine, 101 Dade Road, Yuexiu District, Guangzhou 510120, China; ^2^Department of Cardiology, The Second Clinical Medical School, Yangzhou University, Yangzhou 225001, China; ^3^Animal Laboratory, Guangdong Province Hospital of Chinese Medicine, 2nd Affiliated Hospital of Guangzhou University of Chinese Medicine, Guangzhou 510120, China; ^4^Department of Oral and Maxillary Surgery, Stomatology Hospital of Guangzhou Medical University, Guangzhou 510140, China; ^5^Department of Pharmaceutical Sciences, College of Pharmacy, Northeast Ohio Medical University, Rootstown, OH 44272, USA

## Abstract

*Background*. Cardiac hypertrophy occurs in many cardiovascular diseases. Apocynum tablet (AT), a traditional Chinese medicine, has been widely used in China to treat patients with hypertension. However, the underlying molecular mechanisms of AT on the hypertension-induced cardiac hypertrophy remain elusive. The current study evaluated the effect and mechanisms of AT on cardiac hypertrophy. *Methods*. We created a mouse model of cardiac hypertrophy by inducing pressure overload with surgery of transverse aortic constriction (TAC) and then explored the effect of AT on the development of cardiac hypertrophy using 46 mice in 4 study groups (combinations of AT and TAC). In addition, we evaluated the signaling pathway of phosphorylation of ERK1/2, AKT, and protein expression of GATA4 in the cardioprotective effects of AT using Western blot. *Results*. AT inhibited the phosphorylation of Thr202/Tyr204 sites of ERK1/2, Ser473 site of AKT, and protein expression of GATA4 and significantly inhibited cardiac hypertrophy and cardiac fibrosis at 2 weeks after TAC surgery (*P* < 0.05). *Conclusions*. We experimentally demonstrated that AT inhibits cardiac hypertrophy via suppressing phosphorylation of ERK1/2 and AKT.

## 1. Introduction

Cardiac hypertrophy occurs in many heart diseases (e.g., essential hypertension, myocardial infarction, and valvular diseases). Characterized by an increase in the size of cardiac myocytes and whole heart enlargement, cardiac hypertrophy is an adaptive reaction in response to increased pressure overload. Sustained after-overload usually induces an initial compensatory hypertrophy, which can progress to pathologic cardiac hypertrophy and finally to congestive heart failure [1]. Overpressure is a major initiative stimulus triggering protein synthesis, gene expression reprogramming, and activation of various signaling molecules, such as protein kinase C (PKC) pathway, the mitogen-activated protein kinases (MAPK) pathway, and the phosphatidylinositol 3-kinase (PI3-K)/Akt pathway, and, thus, subsequently modifies transcriptional regulatory factors (GATA4) and resulting in cardiac hypertrophy [2–4].

Apocynum tablet (AT, Guangdong Peace Pharmaceutical Corp, Guangdong, China), a traditional Chinese medicine formulated mainly with following herbs:* Apocynum, Chrysanthemum,* and* Fangchi*, has been widely used in China to treat patients with hypertension [5]. Clinical trials demonstrated that apocynum tablet is effective and safe for treating hypertension [6, 7]. However, the underlying molecular mechanisms of AT on the hypertension-induced cardiac hypertrophy remain elusive. The current study evaluated a hypothesis that AT can protect hypertension patients from cardiac hypertrophy by inhibiting phosphorylation of ERK1/2 and AKT. To evaluate this hypothesis, we compared cardiac hypertrophy and phosphorylation of ERK1/2 and AKT between mouse models of hypertension with and without pretreatment of AT.

## 2. Methods

### 2.1. Animals and Reagents

This study was performed in accordance with the guidelines and with approval from the Institutional Animal Care and Use Committee of Guangdong Province Hospital of Chinese Medicine, Guangzhou University of Traditional Chinese Medicine, and with the Guide for the Care and Use of Laboratory Animals published by the National Academy of Sciences (8th edition, Washington, DC, 2011).

### 2.2. Transverse Aortic Constriction

**Figure 1:**
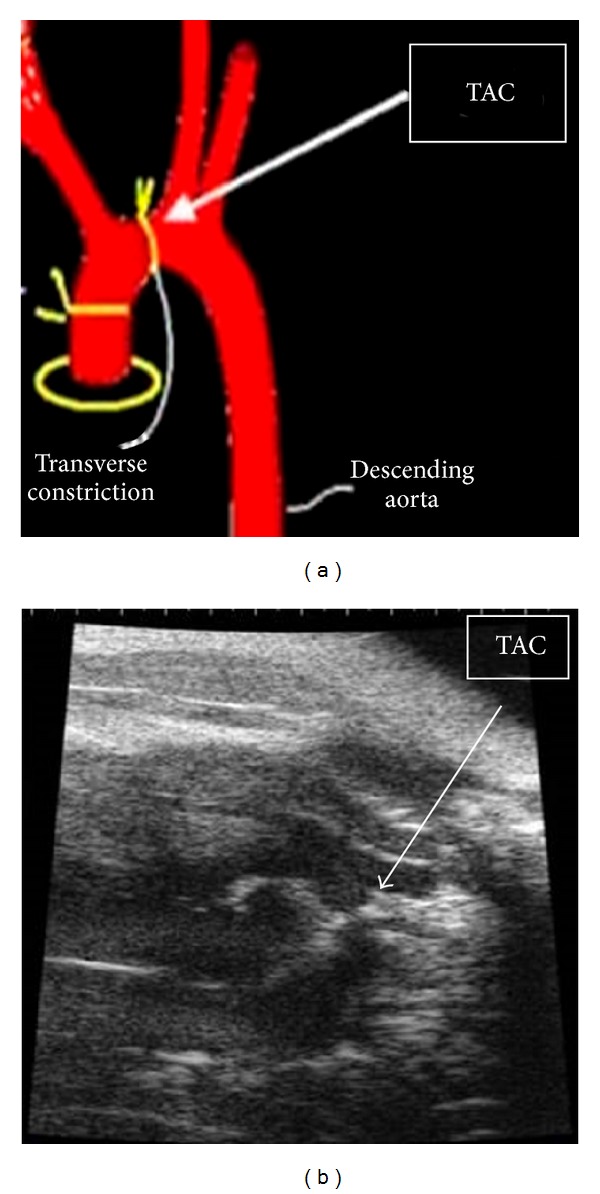
Schematic diagram (a) and echocardiography (b) of TAC surgery.

To explore the effects of AT in cardiac hypertrophy, we constructed a cardiac hypertrophy model by using transverse aortic constriction (TAC) surgery to impose pressure overload in mice using similar protocol as was published previously [8, 9]. In brief, increased pressure in the transverse thoracic aorta was induced by means of TAC (Figures 1(a) and 1(b)). Male mice (C57BL/6J, 8 to 10 weeks old, 25 ± 5 g body weight, from the Experimental Animal Center of Guangdong Province) were anesthetized with pentobarbital sodium (60 mg/kg IP, Sigma-Aldrich Corp). The mice were orally intubated with 20-gauge tubing and ventilated (Harvard Apparatus Rodent Ventilator, model 687) at 110 breaths per minute (0.2 mL tidal volume). A 3 mm center thoracotomy was created. The transverse aortic arch was ligated (7–0 Prolene) between the innominate and left common carotid arteries with an overlying 28-gauge needle, and then the needle was removed, leaving a discrete region of stenosis. The chest was closed, and the pneumothorax was evacuated. Some mice were subjected to a sham operation in which the aortic arch was visualized but not banded.

### 2.3. Protocol

Based on literature, clinical usage (a 70 Kg person taking 2 AT pills each time, three times a day, each tablet weighs 0.6 g), and the Meeh-Rubner equation of dose conversion between humans and mice, human dosage of AT (0.51 g/kg/day) equals 0.67 g/kg/day for mouse. We choose 0.6 g/kg dosage for mice by intragastric administration (i.g) daily. Mice were assigned to four groups: NS-SHAM group, NS-TAC group, AT-SHAM group, and AT-TAC group. Mice in NS-SHAM received saline i.g and all the surgery except constricting the aorta; mice in NS-TAC were subjected to saline i.g and TAC surgery; AT-SHAM mice received AT i.g and all the surgery except constricting the aorta; AT-TAC mice received AT i.g and TAC surgery.

### 2.4. HW Assessment and Histological Examination

At the completion of the experiment, animals were euthanized and their hearts were removed, the left ventricle was quickly separated from the atria and right ventricular free wall, and their heart [left ventricle + right ventricle] weights (HW) and body weights (BW) were determined. Then, left ventricles were fixed overnight in 4% paraformaldehyde before embedding in paraffin. Sections of 5 *μ*m were prepared and stained with hematoxylin-eosin (HE) or Sirius red for evaluation of myocyte hypertrophy and collagen content, respectively.

Cardiomyocytes from LV cross sections were stained with hematoxylin-eosin, and mean values from each mouse were calculated by measurements from 60 to 80 cells from an individual mouse using light microscopy at × 400 magnification. Sirus-stained sections were quantitatively analyzed using light microscopy at × 40 magnification to evaluate myocardial fibrosis using the difference in color (red fibrotic area as opposed to yellow myocardium). Digital photographs were obtained by using a color image analyzer (QWin Colour Binary 1, LEICA).

### 2.5. Western Blot Analysis

Western blot was performed as previously described [10]. Briefly, samples were lysed in 100 *μ*L buffer containing 20 mM Tris-HCl (pH 7.4), 100 mM NaCl, 10 mM sodium pyrophosphate, 5 mM EDTA, 50 mM NaF, 1 mM sodium vanadate, 0.1% SDS, 10% glycerol, 1% Triton X-100, 1% sodium deoxycholate, 1 mM leupeptin, 0.1 mM aprotinin, and 1 mM PMSF. Protein concentration was determined with a BCA protein assay kit (Pierce Biotechnology, Inc, Rockford, IL, USA), and proteins were separated on a 10% SDS-polyacrylamide gel and then electrophoretically transferred to nitrocellulose membranes (Pall Corporation, East Hill, NY, USA). Results are expressed as the changes over control (Con) or sham (SHAM of TAC group). Following antibodies were used in this study: anti-phospho-ERK1/2 (Thr202/Tyr204, Cell Signaling Technology, Beverly, MA, USA), anti-phospho-PKB (Ser473, Cell Signaling Technology), anti-ERK1/2 (Santa Cruz Technology, Delaware, CA, USA), and anti-GATA4 (Selleckchem Technology, Houston, TX, USA). The sheets were analyzed with antibodies according to the supplier's protocol and visualized peroxidase using an enhanced-chemiluminescence system (ECL kit, Pierce Biotechnology, Inc.). Bands were visualized by use of a super western sensitivity chemiluminescence detection system (Pierce, IL). Autoradiographs were quantitated by a densitometry Science Imaging system (Bio-Rad, Hercules, CA).

### 2.6. Statistical Analysis

Data are presented as mean ± SEM. Statistical analysis was performed by one-way analysis of variance followed by Turkey's method or unpaired two-tailed Student's *t*-tests. Results were considered statistically significant at *P* < 0.05.

## 3. Results

### 3.1. AT Inhibited Cardiac Hypertrophy in Response to Pressure Overload

**Figure 2:**
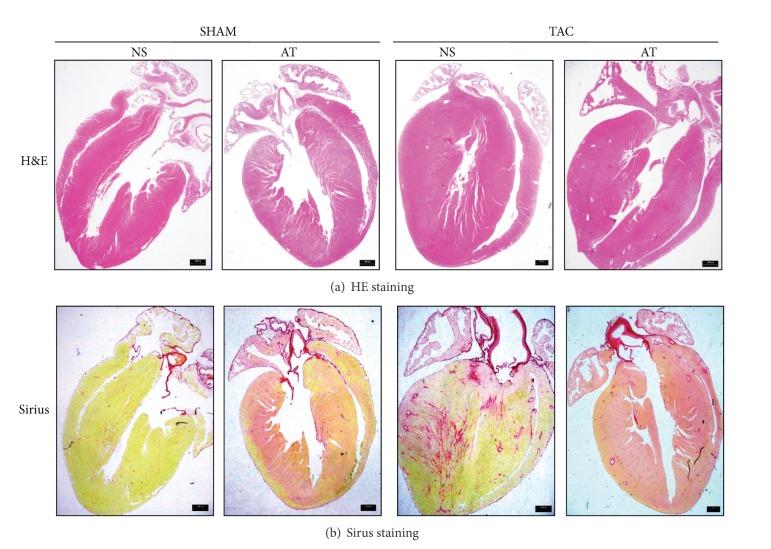
Dye-stained hypertrophic heart sections. (a) H&E-stained (upper) and (b) Sirius red-stained (lower) sections of representative hearts from NS and AT mice 14 days after either SHAM or TAC surgery. Scale at bottom is in mm.

**Figure 3:**
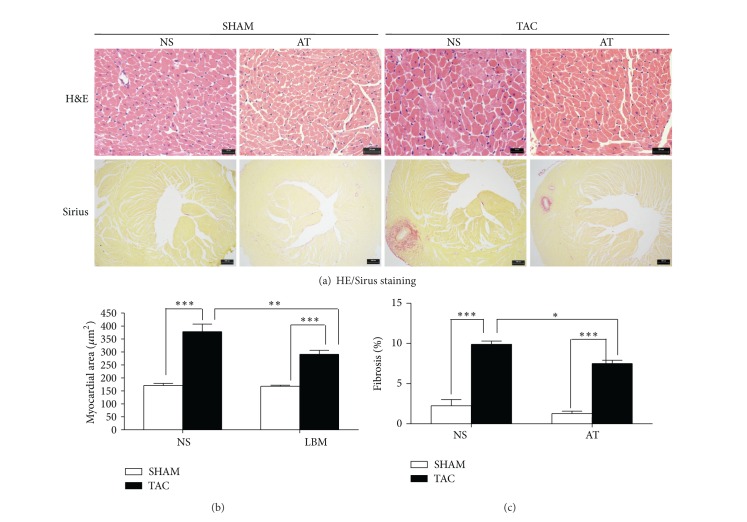
Histological sections of the left ventricular (LV) wall (Groups: NS-SHAM, NS-TAC, AT-SHAN and AT-TAC mice). (a) The LV cross sections of the four groups stained with H&E (×400 magnification, Scale bar, 20 *μ*m) and Sirius red (red staining, ×40 magnifications, Scale bar, 200 *μ*m). (b) Mean cross-sectional area of cardiomyocytes and (c) the fraction of fibrotic area. *P < 0.05, **P < 0.01, and ***P < 0.001, comparison among the groups.

**Figure 4:**
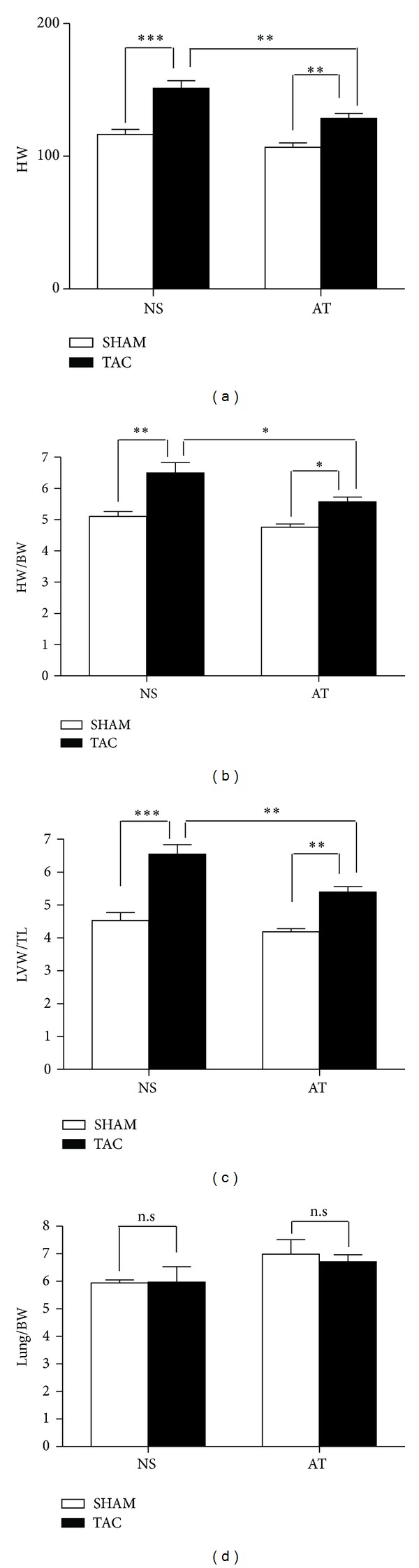
Differences among the four groups. (a) HW, (b) HW/BW, (c) LVW/TL, (d) Lung/BW were compared among the four groups (NS-SHAM, NS-TAC, AT-SHAN, and AT-TAC mice). *P < 0.05, **P < 0.01, and ***P < 0.001; n.s.: no significance.

There were no significant differences in body weight among the four groups of mice (*P* > 0.05, Table 1). At the end of 2 weeks after surgery, cardiomyocytes were much bigger in NS-TAC than NS-SHAM mice (377.8 ± 29.2 *μ*m^2^ versus 170.8 ± 7.8 *μ*m^2^, *P* < 0.001, Figures 2(a), 3(a) and 3(b)). Also cardiac fibrosis formed much more in the NS-TAC mice than in the NS-SHAM mice (9.84 ± 0.42% versus 2.10 ± 0.82%, *P* < 0.001, Figures 2(b) and 3(c)). Heart weights (HW) were significantly heavier in the NS-TAC mice than NS-SHAM mice (HW, 151.2 ± 5.7 mg versus 128.6 ± 3.7 mg, *P* < 0.001, Figure 4(a)). The ratio of left ventricular weight (LVW) to tibal length (TL) was higher in the NS-TAC mice than in the NS-SHAM mice (6.1 ± 0.5 versus 4.5 ± 0.2, *P* < 0.01, Figure 4(c)). However, the ratios of lung weight to body weight (BW) differed insignificantly among the four groups (*P* > 0.05, Figure 4(d)). Therefore, these results showed that compensate pathological cardiac hypertrophy, but not decompensate heart failure, was formed after TAC surgery. Subsequently, we compared the effects between NS-TAC mice and AT-TAC mice. As shown in Figure 4, HW and LVW/TL were significantly lower in AT-TAC (HW, 128.6 ± 3.7 mg; LVW/TL, 5.4 ± 0.2, resp.) than NS-TAC mice (HW, 151.2 ± 5.7 mg, *P* < 0.001; LVW/TL, 6.1 ± 0.5, *P* < 0.001, resp., Figure 4(c)). Together, these date demonstrated that AT could inhibit cardiac hypertrophy in response to pressure overload.

### 3.2. AT Decreased the Mortality in Response to Pressure Overload

**Figure 5:**
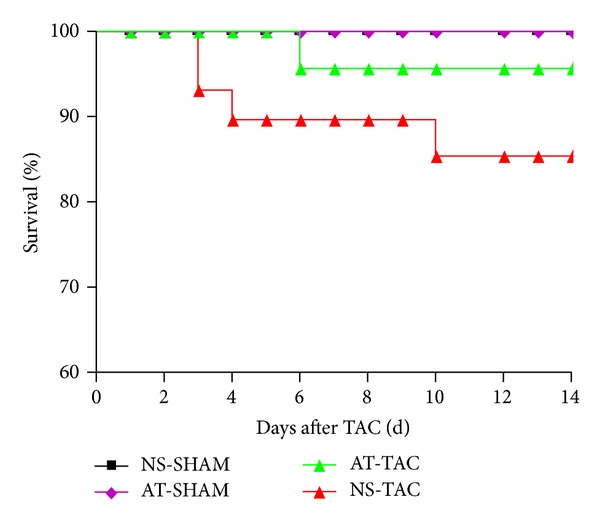
Kaplan-Meier survival curves of NS-SHAM, AT-SHAM, AT-TAC, and NS-TAC mice.

Recent clinical data have demonstrated that AT drastically improved cardiac function, structure, and quality of life in hypertension patients [11]. One critical question arising from the observation that AT prevented hypertrophy in the TAC mice is whether it has a beneficial or harmful impact on animal survival. To investigate this, we evaluated the effects of AT on post-TAC survival by analyzing Kaplan-Meier curves in the four groups of mice. We found that the survival rate was significantly higher in the AT-TAC mice than the NS-TAC mice (Figure 5). AT-TAC mice displayed a significantly improved survival compared to NS-TAC mice (Figure 5), whereas NS-TAC mice had 87% survival (*n* = 31) 2 weeks after TAC, AT-TAC mice had 96% survival (*n* = 28; *P* < 0.001). AT-SHAM and NS-SHAM mice that underwent sham surgery, which included thoracotomy but no constriction of the aorta, had 100% survival for both AT-SHAM and NS-SHAM groups (Figure 5). Thus, AT significantly improved mice survival after TAC surgery in response to pressure overload.

### 3.3. Phosphorylation of ERK1/2 and AKT Were Inhibited after AT Stimulation

**Figure 6:**
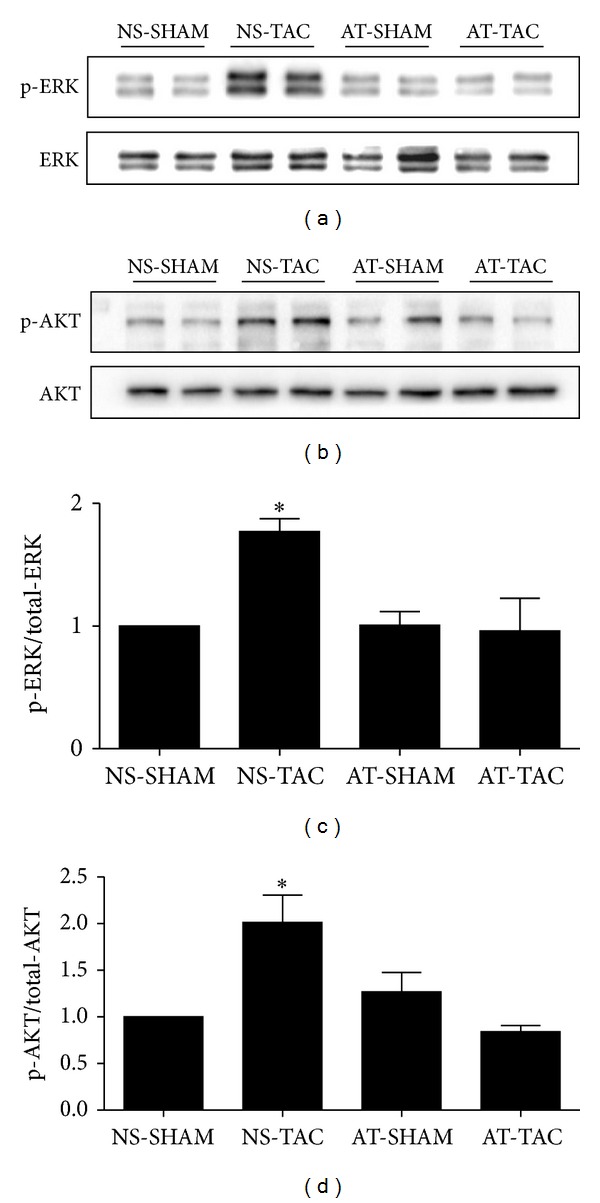
AT inhibited the phosphorylation of ERK1/2 and AKT in response to TAC. (a) Phosphorylated (p)-Thr202/204 extracellular signal-regulated kinase (ERK) 1/2 and (b) p-Ser473 protein kinase B (AKT), and quantified data for (c) p-ERK1/2 and for (d) p-AKT. Data (mean ± SEM, *n* = 3) were expressed as fold changes from total protein (ERK1/2, AKT) and control (NS-SHAM). *P < 0.05 study group versus NS-SHAM group.

To investigate the mechanisms of AT inhibition on cardiac hypertrophy in response to pressure overload, we focused on MAPK and AKT, which are two main signal transduction pathways involved in cardiac hypertrophy [12]. By using Western blot analysis, we found that the ERK1/2 phosphorylations of threonines at 202th and 204th sites were enhanced in NS-TAC group, compared with NS-SHAM group (*P* < 0.05, Figures 6(a) and 6(c)). Interestingly, the phosphorylations of threonines at 202th and 204th sites were reduced after AT stimulation in AT-TAC group, compared with NS-TAC group. Thus, these data revealed that AT reversed TAC-induced cardiac hypertrophy through the ERK1/2 phosphorylations of threonines at 202th and 204th sites. Moreover, we detected that the AKT phosphorylation of tyrosine at 473th site, which was increased in NS-TAC group (*P* < 0.05, versus NS-SHAM groups, Figures 6(b) and 6(d)), but decreased after AT stimulation in AT-TAC group. Together, these data revealed that AT reversed TAC-induced cardiac hypertrophy via suppressing the ERK1/2 phosphorylations of threonines at 202th and 204th sites and AKT phosphorylation of tyrosine at 473th site.

### 3.4. The Protein Expression of GATA4 Was Reduced after AT Treatment in Mice

**Figure 7:**
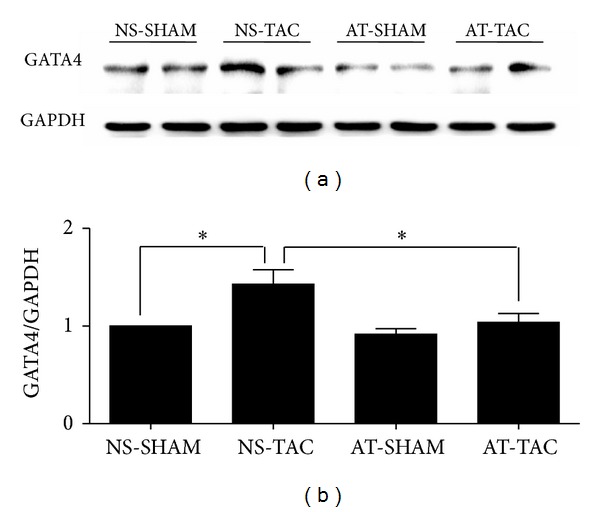
The protein expression of GATA4 was reduced after AT treatment in mice. (a) Western blot bands of the protein expression of GATA4 and GAPDH and (b) their fold changes in the four groups (NS-SHAM, NS-TAC, AT-SHAM, and AT-TAC). Data are mean ± SEM (*n* = 7). *P < 0.05 NS-TAC versus NS-SHAM and AT-TAC groups.

To further investigate the potential role of the ERK1/2 and AKT pathway in the hypertrophic-inhibiting effect of AT in TAC, we analyzed a typical downstream target, GATA4. GATA4 is a zinc finger, containing transcription factor that plays key roles in promoting heart growth and regulating cardiac hypertrophy [13, 14], and is associated with multiple hypertrophic signaling pathways, such as ERK1/2 [15], p38, Akt [16], and CnA/NFATc3 [17]. As shown in Figure 7, protein expression of GATA4 in the NS-TAC group was significantly increased compared with NS-SHAM group (*P* < 0.05), in consistence with literature [18, 19]. However, after AT stimulation, the protein expression of GATA4 was reduced in AT-TAC mice, compared with NS-TAC group (*P* < 0.05, Figures 7(a) and 7(b)), which was also consistent with the changes in ERK and AKT. Thus, these data revealed that AT reversed TAC-induced cardiac hypertrophy through the protein expression of GATA4.

**Figure 8:**
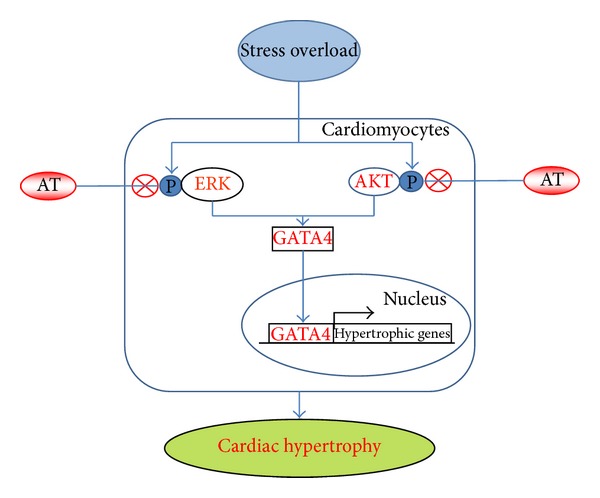
A model of pathways in the cardioprotection of AT in response to pressure stress overload. TAC (stress overload) could activate phosphorylation of the protein kinases of ERK1/2 and AKT, enhance the expression of GATA4, promote the transcription of hypertrophic gene, and result in cardiac hypertrophy and cardiac fibrosis. AT could inhibit the phosphorylation of ERK1/2 and AKT, reduce GATA4, and inhibit pathological development of cardiac hypertrophy. ⨂ denotes inhibition of protein kinase by AT treatment.

We formulated a working model based on the observations of this study (Figure 8). Stress overload of TAC could activate the phosphorylation of the protein kinases of ERK1/2 and AKT, enhance the expression of GATA4, promote the transcription of hypertrophic gene, and result in cardiac hypertrophy and fibrosis. AT could inhibit the phosphorylation of ERK1/2 and AKT, reduce GATA4, and inhibit pathological development of cardiac hypertrophy.

## 4. Discussion

This study illustrated the mechanism of AT protection against pathological cardiac hypertrophy in mice. Our results can be summarized as follows: (1) AT could attenuate cardiac hypertrophy and cardiac fibrosis in response to pressure overload in vivo; (2) the effects of AT could be mediated by mitogen-activated protein kinase 1/2 signaling pathway; (3) AKT signaling pathway also participated in the protective role of AT on pathological cardiac hypertrophy; and (4) GATA4 was also reduced after AT stimulation in response to TAC. To our knowledge, this is the first study to demonstrate the effectiveness and mechanism of AT in reducing pathological cardiac hypertrophy in response to pressure overload in mice.

Clinical studies revealed that systolic and diastolic blood pressure in hypertension patients were reduced more significantly by treatments with apocynum tablets than with nifedipine alone. Apocynum tablet in combination with nifedipine had a stable antihypertensive effect [6, 7]. Apocynum leaves, which are a major ingredient of apocynum tablets, contain three main active compounds: quercetin, flavonoids, and carbohydrates [5]. Quercetin could enhance capillary resistance, reduce capillary fragility, lower blood pressure, dilate coronary artery, and enhance coronary blood flow [20]. Another major ingredient of AT, chrysanthemum, could increase cardiac output and stroke volume and slowly and persistently decrease blood pressure [21]. The current study further advanced our knowledge by demonstrating AT treatment could prevent the development of pathological cardiac hypertrophy.

Cardiac hypertrophy is regulated by a network of signaling pathways, including beta-adrenergic receptor signaling and associated kinases, PKC-alpha, Ca^2+^/calmodulin-dependent kinase II signaling, Phosphodiesterase 5, MAPKs, HDAC, PI3-K/AKT, and GATA4 [22]. Previous studies demonstrated that cardiac hypertrophy is mediated by a PI3-K/AKT and ERK1/2 pathway, which can be pharmacological targets for cardioprotection [23, 24]. Considering there is still no effective Chinese medicine to treat cardiac hypertrophy, we did not set the positive control of Chinese medicine and different AT dosages. The present study demonstrated that AT significantly decreased cardiac hypertrophy and suppressed the increases of phosphorylation of Akt and ERK1/2 following the TAC surgery in mice.

The zinc-finger containing transcription factor GATA4 has been ascribed to a number of critical functions in the heart, spanning from the specification and differentiation of cardiac myocytes early in development to the regulation of the cardiac hypertrophic response in the adult. GATA4 mediates these processes through directly binding to the promoters of the ANF, BNP, alpha-MHC, and beta-MHC genes, thereby controlling their expression in the heart [25]. Overexpression of GATA4 by adenoviral gene transfer induced cardiomyocyte hypertrophy [26]. Cardiac specific knockout of GATA4 in adult mouse renders the heart less able to hypertrophy with agonist or pressure overload stimulation, as well as more likely to succumb to heart failure [27]. Both ERK1/2 and AKT activity were necessary for the increase in GATA4 DNA binding from hearts underwent acute wall stretching [28]. Here we demonstrated that GATA4 expression was reduced after AT treatment in response to pressure overload. Together, our findings contribute to further understanding the molecular mechanisms of cardiac protection of AT.

Although there are several cardioprotective drugs for treating heart failure and cardiac hypertrophy, such as beta-adrenergic receptor blocker, ACE inhibitor, and calcium channel blockers, the mobility and mortality of heart failure and cardiac hypertrophy were still high in the United States [29]. These inadequate results could be due to the presence of multiple mechanistic pathways of cardiac hypertrophy and the lack of therapies targeting these pathways simultaneously. Increasing evidences demonstrated that there are several bioactive ingredients contributing to AT's cardioprotection effects against cardiac hypertrophy, such as apocynum leaves and wild chrysanthemum [30, 31]. The complex profile of active ingredients in AT could act on multiple signaling pathways, which might possibly overcome the deficiencies of these single-target drugs in protecting against cardiac hypertrophy.

We used 28 G needle to construct the TAC model in the current study. This method reliably produced a model of cardiac hypertrophy 2 weeks after TAC surgery (Figure 2). Our preliminary study showed that we produced stable aortic pressure gradient (AoPg) waved in 70–90 mmHg after TAC surgery for 1 week (data not shown), in consistence with a report from Vatner's laboratory [9].

In conclusion, the present results enhanced our understanding of the role of AT on cardiac hypertrophy. We demonstrated that selective ERK1/2 and AKT modulation for cardioprotection is feasible, suggesting their possibilities to be therapeutic targets. These data experimentally provided evidences that AT inhibits cardiac hypertrophy from pressure overload and elucidated the mechanisms of the effective AT treatment in patients with cardiac hypertrophy.

## Figures and Tables

**Table 1:** Anatomical data of the four groups.

Group	NS-SHAM (*n* = 6)	NS-TAC (*n* = 15)	LBM-SHAM (*n* = 9)	LBM-TAC (*n* = 16)
BW, g	23 ± 0.3	23 ± 0.4	22 ± 0.3	23 ± 0.2
HW, mg	116.5 ± 3.8	151.2 ± 5.7∗∗∗	106.6 ± 4.0	128.6 ± 3.7^∗##^
LVW, mg	78.7 ± 3.9	104.5 ± 8.9∗∗	57.7 ± 9.5	92.4 ± 2.9^∗∗#^
Lung, mg	135.7 ± 3.2	147.9 ± 8.7	167.7 ± 10.4	154.6 ± 5.8
Liver, mg	876.0 ± 17.5	1054.6 ± 47.1	948.5 ± 51.7	980.4 ± 33.5
TL, mm	17.4 ± 0.2	17.3 ± 0.1	17.1 ± 0.1	17.1 ± 0.1
HW/BW	5.1 ± 0.2	6.5 ± 0.3∗∗	4.8 ± 0.1	5.6 ± 0.1^#^
HW/TL	6.7 ± 0.2	8.8 ± 0.3∗∗∗	6.2 ± 0.2	7.5 ± 0.2^∗##^
LVW/TL	4.5 ± 0.2	6.1 ± 0.5∗∗	3.4 ± 0.6	5.4 ± 0.2^∗∗#^
Lung/BW	5.9 ± 0.1	6.4 ± 0.5	7.6 ± 0.6	6.7 ± 0.3
Liver/BW	38.4 ± 0.6	48.9 ± 2.4	42.3 ± 1.7	42.5 ± 1.5

NS: saline; AT: apocynum tablets; TAC: transverse aortic constriction; BW: body weight; HW: heart weight; LVW: left ventricular weight; TL: tibial length; ∗*P* < 0.05, ∗∗*P* < 0.01, and ∗∗∗*P* < 0.001 compared to NS-SHAM or AT-SHAM from the same group. ^#^ *P* < 0.05, ^##^ *P* < 0.01, compared to AT-TAC from NS-TAC group.

## References

[1] van Berlo JH, Maillet M, Molkentin JD (2013). Signaling effectors underlying pathologic growth and remodeling of the heart. *Journal of Clinical Investigation*.

[2] Kandalam V, Basu R, Moore L (2011). Lack of tissue inhibitor of metalloproteinases 2 leads to exacerbated left ventricular dysfunction and adverse extracellular matrix remodeling in response to biomechanical stress. *Circulation*.

[3] Hu Y, Matkovich SJ, Hecker PA, Zhang Y, Edwards JR, Dorn GW (2012). Epitranscriptional orchestration of genetic reprogramming is an emergent property of stress-regulated cardiac microRNAs. *Proceedings of the National Academy of Sciences of the United States of America*.

[4] Dionyssiou MG, Nowacki NB, Hashemi S (2013). Cross-talk between glycogen synthase kinase 3*β* (GSK3*β*) and p38MAPK regulates myocyte enhancer factor 2 (MEF2) activity in skeletal and cardiac muscle. *Journal of Molecular and Cellular Cardiology*.

[5] Liu HQ (2004). Determination of isoquercitrin of compound kendir leaves tablets HPLC. *Traditional Chinese Medicine*.

[6] Xu Y (2010). Clinical observation of Apocynum tablets combined with nifedipine for treating 77 patients of essential hypertension. *Journal of Cardiovascular and Pulmonary Diseases*.

[7] Wang SH (2011). Nifedipine combined with apocynum tablets for treating essential hypertension. *Asia Pacific Traditional Medicine*.

[8] Qi JY, Xu M, Lu ZZ, Zhang YY (2009). Differential expression of 14-3-3ε during physiological, pathological cardiac hypertrophy and chronic heart failure in mice. *Gene Therapy and Molecular Biology*.

[9] Qiu H, Lizano P, Laure L (2011). H11 kinase/heat shock protein 22 deletion impairs both nuclear and mitochondrial functions of stat3 and accelerates the transition into heart failure on cardiac overload. *Circulation*.

[10] Qi J-Y, Xu M, Lu Z-Z, Zhang Y-Y (2010). 14-3-3 inhibits insulin-like growth factor-I-induced proliferation of cardiac fibroblasts via a phosphatidylinositol 3-kinase-dependent pathway. *Clinical and Experimental Pharmacology and Physiology*.

[11] Li PP, Zhao LS, Cui SX (2011). Influence of Apocynum tablet on ambulatory blood pressure in elderly patients with hypertension. *Chinese Journal of Gerontology*.

[12] Meng R, Pei Z, Zhang A (2011). AMPK activation enhances PPAR*α* activity to inhibit cardiac hypertrophy via ERK1/2 MAPK signaling pathway. *Archives of Biochemistry and Biophysics*.

[13] Xin M, Olson EN, Bassel-Duby R (2013). Mending broken hearts: cardiac development as a basis for adult heart regeneration and repair. *Nature Reviews Molecular Cell Biology*.

[14] Van Berlo JH, Elrod JW, Aronow BJ, Pu WT, Molkentin JD (2011). Serine 105 phosphorylation of transcription factor GATA4 is necessary for stress-induced cardiac hypertrophy in vivo. *Proceedings of the National Academy of Sciences of the United States of America*.

[15] Kehat I, Davis J, Tiburcy M (2011). Extracellular signal-regulated kinases 1 and 2 regulate the balance between eccentric and concentric cardiac growth. *Circulation Research*.

[16] Oudit GY, Penninger JM (2009). Cardiac regulation by phosphoinositide 3-kinases and PTEN. *Cardiovascular Research*.

[17] Wang K, Long B, Zhou J, Li P (2010). miR-9 and NFATc3 regulate myocardin in cardiac hypertrophy. *The Journal of Biological Chemistry*.

[18] van Berlo JH, Aronow BJ, Molkentin JD (2013). Parsing the roles of the transcription factors GATA-4 and GATA-6 in the adult cardiac hypertrophic response. *PLoS ONE*.

[19] Kobayashi S, Mao K, Zheng H (2007). Diminished GATA4 protein levels contribute to hyperglycemia-induced cardiomyocyte injury. *The Journal of Biological Chemistry*.

[20] Kwan C, Zhang W, Nishibe S, Seo S (2005). A novel in vitro endothelium-dependent vascular relaxant effect of *Apocynum venetum* leaf extract. *Clinical and Experimental Pharmacology and Physiology*.

[21] Wu DH, Yang LW, Su WW (2004). The progress in chemical constituents and pharmacological studies of wild chrysanthemum. *Chinese Herbal Medicine*.

[22] Burchfield JS, Xie M, Hill JA (2013). Pathological ventricular remodeling: mechanisms: part 1 of 2. *Circulation*.

[23] Pillai VB, Sundaresan NR, Gupta MP (2014). Regulation of Akt signaling by sirtuins: its implication in cardiac hypertrophy and aging. *Circulation Research*.

[24] Ulm S, Liu W, Zi M (2014). Targeted deletion of ERK2 in cardiomyocytes attenuates hypertrophic response but provokes pathological stress induced cardiac dysfunction. *Journal of Molecular and Cellular Cardiology*.

[25] Molkentin JD (2000). The zinc finger-containing transcription factors GATA-4, -5, and -6: ubiquitously expressed regulators of tissue-specific gene expression. *The Journal of Biological Chemistry*.

[26] Liang Q, de Windt LJ, Witt SA, Kimball TR, Markham BE, Molkentin JD (2001). The transcription factors GATA4 and GATA6 regulate cardiomyocyte hypertrophy *in vitro* and *in vivo*. *The Journal of Biological Chemistry*.

[27] Oka T, Maillet M, Watt AJ (2006). Cardiac-specific deletion of gata4 reveals its requirement for hypertrophy, compensation, and myocyte viability. *Circulation Research*.

[28] Tenhunen O, Sármán B, Kerkelä R (2004). Mitogen-activated protein kinases p38 and ERK 1/2 mediate the wall stress-induced activation of GATA-4 binding in adult heart. *The Journal of Biological Chemistry*.

[29] Go AS, Mozaffarian D, Roger VL (2014). Heart disease and stroke statistics—2014 update: a report from the American Heart Association. *Circulation*.

[30] Yang H, Xie F, Yang Y, Luo Y (2011). Preparation and in vitro release characteristics of pulsed-release tablets of *Apocynum venetum*. *Zhongguo Zhong Yao Za Zhi*.

[31] Wu Q, Chen CX, Gu WL, Gao JP, Wan Y, Lv J (2010). Effect of Chrysanthemum indicum on ventricular remodeling in rats. *Zhong Yao Cai*.

